# Free Water‐Corrected Fractional Anisotropy in Normal‐Appearing White Matter as a Potential Neuroimaging Biomarker for Attention and Executive Function Impairment in Cerebral Small Vessel Disease

**DOI:** 10.1111/cns.70475

**Published:** 2025-06-09

**Authors:** Qingyang Fu, Yage Qiu, Ying Hu, Yuanzheng Wang, Yao Wang, Wentao Hu, Qun Xu, Yawen Sun, Yan Zhou

**Affiliations:** ^1^ Department of Radiology, Ren Ji Hospital, School of Medicine Shanghai Jiao Tong University Shanghai China; ^2^ Department of Neurology, Ren Ji Hospital, School of Medicine Shanghai Jiao Tong University Shanghai China; ^3^ Renji‐UNSW CHeBA Neurocognitive Center, Ren Ji Hospital, School of Medicine Shanghai Jiao Tong University Shanghai China; ^4^ Department of Health Manage Center, Ren Ji Hospital, School of Medicine Shanghai Jiao Tong University Shanghai China

**Keywords:** attention/executive function, cerebral small vessel disease, free water‐corrected fractional anisotropy, frontal white matter tracts, normal‐appearing white matter

## Abstract

**Objective:**

To investigate the relationship between white matter integrity changes and attention/executive function in cerebral small vessel disease (CSVD), focusing on tract‐specific alterations over stages and identifying key neuroimaging markers affecting cognitive decline.

**Methods:**

A total of 170 CSVD patients, including 103 Vascular Cognitive Impairment (VaMCI) and 67 with no cognitive impairment (NCI) underwent MRI and neuropsychological assessments. Neuroimaging metrics included quantitative susceptibility (QS), free water (FW), FW‐corrected fractional anisotropy (FAt) and FW‐corrected mean diffusivity (MDt) in white matter hyperintensities (WMH) and normal‐appearing white matter (NAWM). WMH volume, gray/white matter volume, lacunar infarcts (LI) volume and counts were also included. Partial correlations were conducted to assess neuroimaging‐cognition relationships, and random forest analysis was employed to determine the relative importance of these indices, with a particular focus on white matter tracts.

**Results:**

The VaMCI group exhibited decreased NAWM FAt and white matter volume, increased NAWM QS, WMH volume, LI volume and counts when compared to the NCI group. NAWM/WMH FAt positively associated with attention/executive function, whereas NAWM/WMH QS, FW, MDt, WMH volume, and LI metrics negatively correlated. Notably, NAWM FAt was the most significant variable, especially in frontal white matter tracts and thalamic radiation.

**Conclusion:**

NAWM FAt significantly impacts attention/executive function in CSVD, particularly in the frontal lobe and thalamic radiation, and may serve as an early potential neuroimaging biomarker for cognitive decline.

## Graphical abstract

In CSVD, NAWM FAt, distinct from early‐stage MRI indices, significantly contributes to attention/executive function decline in the right anterior corona radiata, left superior frontal blade, right superior corona radiata, left anterior limb of internal capsule, and right tapetum.

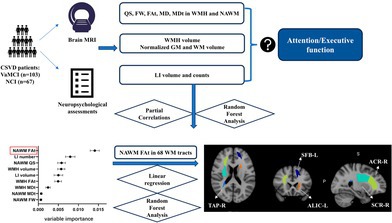

## 1 Introduction

Cerebral small vessel disease (CSVD) is a significant contributor to cognitive decline in the elderly [1]. Numerous neuroimaging studies have been conducted on CSVD. The imaging features of CSVD seen on conventional MRI encompass recent small subcortical infarcts, white matter hyperintensities (WMH), lacunar infarcts (LI), cerebral microbleeds, enlarged perivascular spaces, and cerebral atrophy [2]. Among them, WMH is the most common and prominent feature and is regarded as a typical sign of CSVD. In CSVD, brain damage occurs not only in visible lesions such as WMH but also in the surrounding normal‐appearing white matter (NAWM), and the integrity of white matter is related to cognition [3]. Previous research has demonstrated that white matter alterations are key components of cerebrovascular disease that significantly impact cognitive function [4].

Conventional MRI metrics used to assess white matter injury in CSVD include WMH volume and diffusion tensor imaging (DTI) metrics such as fractional anisotropy (FA) and mean diffusivity (MD), all of which have been linked to the decline in cognitive function [5, 6]. Besides, the volume and numbers of LI are also associated with cognitive decline in CSVD [7]. However, WMH volume is a relatively crude marker, while commonly used, it provides limited information regarding underlying pathophysiological processes. Furthermore, conventional DTI metrics are prone to being influenced by partial volume effects attributed to edema and cerebrospinal fluid [8].

Recently, emerging MRI biomarkers of white matter integrity demonstrate significant associations with cognitive decline. Quantitative susceptibility mapping (QSM) effectively detects paramagnetic iron deposits in gray matter. Recent studies indicated cerebral iron overload may drive cognitive decline in advanced CSVD [9]. However, most studies using QSM predominantly focused on deep gray matter, resulting in a relative scarcity of analyses pertaining to white matter [9, 10]. The myelin sheath serves as the primary contributor to susceptibility‐based contrast in white matter, attributed to its inherent diamagnetic characteristics. Demyelination has been identified as one of the underlying mechanisms of WMH, and myelin loss in NAWM drives cognitive impairment in CSVD [11, 12]. The myelin sheath serves as the primary contributor to susceptibility‐based contrast in white matter, attributed to its inherent diamagnetic characteristics; thus, QSM can be used as a non‐invasive technique for assessing white matter integrity. Our recent studies suggested that axonal loss in the NAWM in the prefrontal lobe and its distinct regional susceptibility distribution patterns were associated with cognitive performance during the progression of CSVD [13, 14]. Therefore, Quantitative susceptibility (QS) is an important indicator reflecting white matter injury in CSVD.

Free water (FW)‐mapping, an advanced DTI technique based on a bi‐tensor model, characterizes tissue properties through the quantification of isotropically unconstrained water [15]. Elevated extracellular fluid content, as reflected by increased FW values, has been consistently associated with neuroinflammatory processes and neurodegenerative changes. FW‐corrected DTI measurements represent the microstructural components of tissue compartments, so that the sensitivity of conventional FA and MD is improved, emphasizing the utility of correcting for free water when assessing white matter integrity [16]. Elevated FW in CSVD was associated with accelerated white matter damage, cognitive decline, and glymphatic dysfunction, suggesting its role as a biomarker for CSVD progression and neurodegeneration [17, 18, 19]. FW‐corrected fractional anisotropy (FAt) was found to be associated with deep medullary vein integrity in subcortical ischemic stroke, highlighting its role in reflecting microstructural white matter injury linked to venous disruption and interstitial diffusivity in CSVD [20]. Furthermore, higher lesional FAt at baseline correlated with greater lesion size reduction in subcortical ischemic stroke over time [21]. Accordingly, FW and FAt may serve as critical MRI indicators reflecting white matter injury in CSVD.

The pathological alterations of white matter injury in CSVD primarily encompass inflammatory, demyelination, and axonal loss, which constitute neurodegenerative processes. Previous studies have demonstrated that FW and QS may be closely associated with early inflammatory changes and subsequent demyelination in CSVD, while FA/FAt predominantly reflects the characteristic axonal loss during the intermediate stage of disease progression. Notably, the NAWM adjacent to WMH undergoes a chronic and progressive transition toward WMH, signifying the advancement of CSVD to its late stage [22, 23, 24]. Baseline WMH burden and its progression dynamically correlated with CSVD evolution and executive dysfunction, furthermore, progressive WMH burden mediated the transition from mild cognitive impairment to dementia [25, 26]. One prior research found that incident LIs demonstrated significant associations with mild deficits in processing speed and executive function in the normal aging [27]. However, the specific brain alterations mentioned above that exert the most significant impact on cognition in CSVD remain unclear.

Our primary goal was to investigate the relationship between neuroimaging changes from early to later stages indicating white matter injury and cognitive function, focusing on attention/executive function, which was impaired early in CSVD [28, 29]. We sought to determine the significance of neuroimaging metrics in relation to attention/executive function, with a specific emphasis on white matter tracts. Based on the pathophysiological process of white matter changes in CSVD, our hypothesis posited that changes in white matter integrity, particularly those occurring in later stages rather than early stages, may play a vital role in the decline of cognitive function. Based on the significance of frontal‐subcortical circuits [30], we proposed that alterations in the microstructure in certain white matter fibers, particularly those located in the frontal region, might contribute to impairment in attention/executive function.

## 2 Materials and Methods

### 2.1 Participants

The study was approved by the Research Ethics Committee of Renji Hospital, School of Medicine, Shanghai Jiao Tong University. All subjects provided written informed consent, and all procedures complied with institutional guidelines.

All CSVD patients were recruited from the Neurology Department of Renji Hospital. Inclusion criteria included: (1) age from 50 to 85; (2) education years ≥ 6; (3) at least a month after the last clinical stroke; (4) presence of at least 1 subcortical lacunar infarction and WMH on MRI; (5) Modified Rankin score ≤ 3 points; (6) capable of completing MRI and relevant neuropsychological tests; (7) willing to sign the informed consent. Exclusion criteria were listed as follows: (1) WMH attributed to non‐vascular factors; (2) acute or chronic cerebral infarction or intracranial space‐occupying lesions; (3) other specific diseases that cause cognitive dysfunction; (4) severe depression (Hamilton Depression Rating Scale score ≥ 24); (5) severe cerebral atrophy; (6) diagnosed with cardioembolic or large‐vessel diseases; (7) history of alcoholism and drug abuse; (8) inability to complete cognitive assessments or MRI scans; (9) history of craniocerebral surgery or brain trauma; (10) contraindications to MR examinations.

Finally, 170 CSVD patients were included in this study, all of whom were right‐handed. Enrolled patients were divided into two groups—Vascular Mild Cognitive Impairment (VaMCI) and no cognitive impairment (NCI) group. VaMCI was diagnosed according to vascular behavioral and cognitive disorders VASCOG criteria, which conformed to the fifth revision of the Diagnostic and Statistical Manual (DSM‐5) [31]. Patients in the VaMCI group exhibited impairments in one or more cognitive domains and did not meet the diagnostic criteria for dementia. Participants were classified as NCI if they had no impairment in cognition.

Based on the above, 170 CSVD patients were divided into VaMCI (*n* = 103) and NCI (*n* = 67) groups.

### 2.2 Neuropsychological Assessment

All participants underwent a battery of standardized cognitive tests within a week after the MRI examination carried out by two trained neuropsychologists. The following tests in Table 1 were used to evaluate general cognitive function and four main cognitive domains.

**TABLE 1:** Neuropsychological tests on cognitive domains.

Cognitive function	Neuropsychological tests
General cognitive function	MoCA, MMSE
Attention/executive function	TMT‐A, TMT‐B, Stroop C‐T, VFT
Memory function	AVLT‐short, AVLT‐long
Language function	BNT
Visuospatial function	Rey‐O copy

Abbreviations: AVLT‐short, AVLT‐long, Rey Auditory Verbal Learning Test of short‐ and long‐delay free recall; BNT, Boston naming test (30 items); MMSE, Mini‐Mental State Examination; MoCA, Montreal Cognitive Assessment; Rey‐O copy, Rey‐Osterrieth Complex Figure Test (copy); Stroop C‐T, Stroop color‐word test C; TMT‐A, TMT‐B, Trail‐Making Tests A and B; VFT, category Verbal Fluency Test.

To evaluate attention and executive function, the raw score of a single neuropsychological test was transformed to a *Z*‐score. The formula is *Z* = (*X*−*M*) /SD. *X* stands for the raw score of each test, *M* stands for the mean value, and SD stands for standard deviation. A composite score was created to represent the performance in the attention/executive domain. In tests where lower scores denoted better performance, the single *Z* score was multiplied by −1 before adding, and the composite scores were divided by the number of tests. The *Z* score for attention/executive function = (−*Z*TMTA−*Z*TMTB−*Z*StroopC + *Z*VFT)/4. Higher *Z* scores indicated better cognitive performance.

### 2.3 MRI Data Acquisition

MRI scanning was conducted on a 3.0T MRI scanner (GE Signal HDxt 3.0 T, USA). Head movement was minimized by employing restraint foam pads. There were no modifications to the hardware or software of the MRI system throughout the study. The acquisition parameters were as follows: (1) Sagittal T1‐weighted (T1w) images were captured with a 3D‐fast spoiled gradient recalled echo (SPGR) sequence: TE = 1.7 ms, TR = 5.5 ms, TI = 450 ms, FA = 15°, FOV = 256 × 256 mm^2^, matrix = 256 × 256, gap = 0, slices = 155, and slice thickness = 1.0 mm; (2) Axial T2‐fluid attenuated inversion recovery sequence (FLAIR): TE = 150 ms, TR = 9075 ms, TI = 2250 ms, FOV = 256 × 256 mm^2^, matrix = 256 × 256, slices = 66, and slice thickness = 2 mm; (3) DTI images were acquired using a spin‐echo single shot echo‐planar pulse sequence: TE = 89.8 ms, TR = 17,000 ms, FOV = 256 × 256 mm^2^, matrix = 128 × 128, gap = 0, slices = 66, slice thickness = 2 mm, 20 diffusion‐weighted scans with a b‐value of 1000 s/mm^2^, and b0 = 0; (4) QSM images with whole‐brain coverage were attained applying a standard flow compensated three‐dimensional spoiled gradient recalled (3D‐SPGR) sequence: TE_1_/ΔTE/TE_16_ = 3.2/2.42/39.5 ms, TR = 42.5 ms, FA = 12°, bandwidth = 62.5 kHz, FOV = 220 × 220 mm^2^, matrix = 256 × 256, slices = 66, slice thickness = 2 mm, and spatial resolution = 0.86 × 0.86 × 1 mm^3^.

### 2.4 MRI Data Processing

**FIGURE 1:**
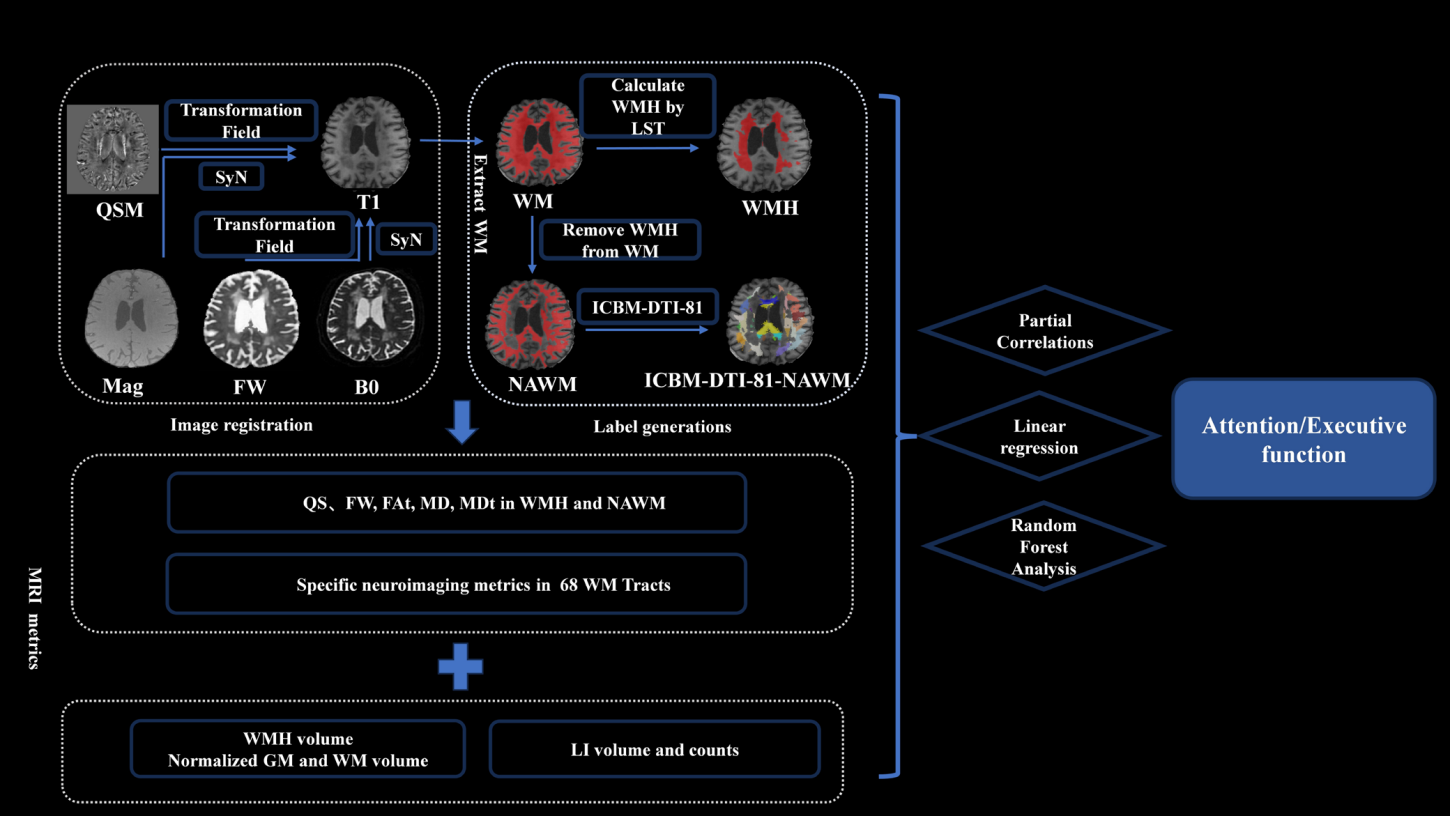
The flowchart showing data processing pipeline and the subsequent statistical analysis. After the reconstruction of QSM images and diffusion MRI preprocessing, Image registration was performed using T1w image as intermedium. Finally, WMH label, NAWM label, and ICBM‐DTI‐81‐NAWM labels were derived. Partial correlations, linear regression and random forest analysis were employed to assess neuroimaging‐cognition relationships, focusing on white matter tracts. FW, free water; ICBM, International Consortium of Brain Mapping; LI, lacunar infarcts; LST, lesion segmentation tool algorithms; Mag, magnitude image; NAWM, normal‐appearing white matter; QSM, quantitative susceptibility mapping; Syn, SyN registration; WM, white matter; WMH, white matter hyperintense.

The post‐processing of data in this study has been demonstrated in previous research [13], including QSM reconstruction, diffusion MRI preprocessing, image registration, and the generation of labels. The processing workflow of the current study is outlined as follows (Figure 1).

#### 2.4.1 QSM Reconstruction

The reconstruction of QSM images was performed in use of the STISuite toolbox (https://people.eecs.berkeley.edu/~chunlei.liu/software.html). The details of QSM processing have been presented in previous studies [32, 33]. The process involved several steps: initially, we applied a Laplacian‐based unwrapping technique to convert the 3D phase image from the complex GRE data. Following this, we utilized the V_SHARP technique to clear the background phase and the STAR‐QSM algorithm to minimize stripe artifacts. Ultimately, we determined the magnetic susceptibility values for dipole inversion, again using the STAR‐QSM.

#### 2.4.2 Diffusion MRI Preprocessing

Before proceeding, we verified that all the diffusion MRI data acquired from various diffusion directions were free of severe artifacts, including but not limited to gross geometric distortion, signal dropout, and bulk motion. To correct for eddy current distortions, we applied the FLIRT (FMRIB's Linear Image Registration Tool) from the FSL (FMRIB Software Library version 5.0.9) as part of the preprocessing steps. FA and MD maps were generated in use of the DTIFIT tool implemented in FSL. FW maps were constructed by fitting a bi‐tensor model based on the diffusion measurements. The comprehensive set of FW maps consists of FW, FA, FAt, MD, and MDt maps.

#### 2.4.3 Image Registration and Generation Labels

T1w image served as intermedium to register all multimodal images into the same space. The magnitude image was aligned with the T1w image through SyN registration. The deformation field was then applied to perform the QSM image and T1w image registration. Similarly, using SyN registration, we performed the B0 image and T1w image registration; subsequently, the deformation field was applied to align the FW, FA, FAt, MD, and MDt images with the T1w image. Furthermore, we registered each T1w image to the MNI (Montreal Neurological Institute, MNI) standard space using the same registration. We used the International Consortium of Brain Mapping (ICBM)‐DTI‐81 white matter (WM) label atlas as a structural template to parcel the white matter into 68 ROI. The inverse deformation field was employed to register ICBM‐DTI‐81 labels from the MNI standard space to individual T1w space.

We used the FAST tool (FMRIB's Automated Segmentation Tool) in FSL to segment white matter templates based on individual T1w images. Next, based on T1w and FLAIR images of each individual, the LST algorithm was used to obtain WMH labels from T1w space [34]. By subtracting the WMH from the white matter template, we derived the label of NAWM. This NAWM label was then combined with the ICBM‐DTI‐81 template to create the ICBM‐DTI‐81‐NAWM label, along with the NAWM distribution across each fiber bundle. We ultimately achieved the WMH label, NAWM label, and ICBM‐DTI‐81‐NAWM labels. Finally, we computed the mean values of the ROIs for the T1w, QSM, and FW maps corresponding to the WMH, NAWM, and ICBM‐DTI‐81‐NAWM labels, and extracted the relevant parameters for subsequent statistical analysis.

In addition, normalized gray and white matter volume, LI volume, and counts were automatically calculated based on MATLAB‐implemented algorithms.

### 2.5 Statistical Analysis

Demographic, clinical, neuroimaging, neuropsychological data were analyzed using SPSS v25 (IBM, Armonk, NY, USA). The following MRI indices were included in the analyses: QS, FW, FAt, MDt in WMH and NAWM, gray and white matter volume, WMH volume, LI volume and counts. The Kolmogorov–Smirnov test was used to examine the normality of the data distribution. Student *t* test was used for normally distributed data, whereas Mann–Whitney *U* test was performed for non‐normally distributed data. Partial correlation analysis was then carried out to investigate correlations between MRI indices and *Z*‐score for attention/executive function, with age, gender ratio, education years, and relevant risk factors as covariates. Random forest regression was applied to elucidate the relative importance of different neuroimaging indices for cognition in the attention/executive domain. This analysis was conducted using a MATLAB‐implemented random forest regression algorithm for modeling; five‐fold cross validation was used during model training, all hyperparameters of the random forest analysis were fixed, and the variable importance was calculated from 100 repetitions. Random forest analysis is a common analytical method used in prior research [35]. Additionally, linear regression was performed to assess the association between specific MRI index in white matter tracts and attention/executive function. The subsequent random forest algorithm was reapplied to assess the importance of imaging variables in a tract‐specific manner. Statistical significance was defined as *p* < 0.05 (without multiple comparison correction).

## 3 Results

### 3.1 Demographic and Clinical Characteristics

Comparisons of demographics and clinical characteristics between two groups are summarized in Table 2.

**TABLE 2:** Demographic and clinical characteristics of the participants.

	VaMCI	NCI	*p* ^a^
Age (mean ± SD)	65.17 ± 6.65	64.37 ± 7.06	0.74^b^
Gender ratio (female %)	26.21%	14.93%	0.08
Education years (mean ± SD)	10.07 ± 2.73	11.66 ± 2.94	< 0.001***
Clinical characteristics			
Hypertension (%)	73.79%	68.66%	0.47
Diabetes mellitus (%)	35.92%	25.37%	0.15
Hyperlipidemia (%)	14.56%	10.45%	0.60
Hypercholesterolemia (%)	14.56%	11.94%	0.62
Current smoker (%)	45.63%	58.21%	0.11
Coronary heart disease (%)	0.97%	5.97%	0.06
Alcohol intake (%)	24.27%	29.85%	0.42

Abbreviations: NCI, no cognitive impairment; SD, standard deviation; VaMCI, vascular mild cognitive impairment.

^a^ Unless otherwise indicated, *p* values were calculated with the Mann–Whitney *U* test.

^b^ Unless otherwise indicated, *p* values were calculated with Student *t* test.

*p‐value < 0.05 was considered to be statistically significant. **p < 0.01, ***p < 0.001.

### 3.2 Group Differences in Neuropsychological Tests

Comparisons between VaMCI and NCI group of neuropsychological tests were shown in Table 3. The results indicated statistically significant differences across all neuropsychological tests and cognitive domains evaluated, highlighting distinct cognitive profiles between individuals with VaMCI and NCI.

**TABLE 3:** Neuropsychological test results in VaMCI and NCI group.

	VaMCI (mean ± SD)	NCI (mean ± SD)	*p* ^a^
General cognitive function			
MoCA	21.40 ± 3.67	26.91 ± 1.45	< 0.001***
MMSE	26.56 ± 1.92	28.52 ± 1.28	< 0.001***
Attention/executive function			
TMT‐A	99.50 ± 44.92	57.69 ± 18.19	< 0.001***
TMT‐B	244.20 ± 99.75	143.94 ± 43.10	< 0.001***
Stroop C‐T	124.86 ± 52.83	80.96 ± 15.41	< 0.001***
VFT	13.12 ± 3.90	16.22 ± 3.60	< 0.001***
*Z*‐score	−0.37 ± 0.72	0.57 ± 0.32	< 0.001***
Memory			
AVLT‐short	3.87 ± 1.99	6.60 ± 2.01	< 0.001***
AVLT‐long	3.03 ± 2.06	6.36 ± 2.29	< 0.001***
Language			
BNT	21.58 ± 3.61	25.70 ± 2.50	< 0.001***
Visuospatial function			
Rey‐O copy	31.62 ± 5.99	35.10 ± 1.53	< 0.001***

Abbreviations: AVLT‐short, AVLT‐long, Rey Auditory Verbal Learning Test of short‐ and long‐delay free recall; BNT, Boston naming test (30 items); MMSE, Mini‐Mental State Examination; MoCA, Montreal Cognitive Assessment; NCI, no cognitive impairment; Rey‐O copy, Rey‐Osterrieth Complex Figure Test (copy); SD, standard deviation; Stroop C‐T, Stroop color‐word test C; TMT‐A, TMT‐B, Trail‐Making Tests A and B; VaMCI, Vascular Mild Cognitive Impairment; VFT, category Verbal Fluency Test.

^a^ *p* value was calculated with the Mann–Whitney *U* test.

*p‐value < 0.05 was considered to be statistically significant. **p < 0.01, ***p < 0.001.

### 3.3 Group Differences Between VaMCI and NCI in MRI Indices

As shown in Table 4, in the VaMCI group, NAWM FAt was significantly lower, while WMH volume, NAWM QS, LI volume, and counts were significantly higher compared to the NCI group.

**TABLE 4:** MRI indices in VaMCI group and NCI group.

MRI indices	VaMCI (mean ± SD)	NCI (mean ± SD)	*p* ^a^
NAWM QS (ppm)	−0.0007 ± 0.0006	−0.0008 ± 0.0005	0.039*^,^ ^b^
NAWM FW	0.3400 ± 0.1985	0.3388 ± 0.1982	0.992^b^
NAWM FAt	0.4048 ± 0.0267	0.4225 ± 0.0484	0.007**
NAWM MDt (mm^2^/s)	0.0007 ± 0.0006	0.0006 ± 0.0006	0.235
WMH QS (ppm)	−0.0085 ± 0.0071	−0.0080 ± 0.0080	0.839
WMH FW	0.3677 ± 0.0261	0.3651 ± 0.0373	0.232
WMH FAt	0.5106 ± 0.1031	0.5244 ± 0.1272	0.356^b^
WMH MDt (mm^2^/s)	0.0007 ± 0.0001	0.0007 ± 0.0001	0.085
WMH volume (cm^3^)	8.74 ± 11.92	4.93 ± 6.99	0.010^c^
GM volume (cm^3^)	568.09 ± 71.55	599.14 ± 58.60	0.088^b^
WM volume (cm^3^)	475.61 ± 58.27	487.51 ± 48.30	0.091^b^
LI volume (cm^3^)	0.2467 ± 0.3458	0.1220 ± 0.1891	0.003**
LI counts	3.22 ± 3.96	1.65 ± 2.48	0.002**

Abbreviations: FAt, FW‐corrected fractional anisotropy; FW, free water; GM, gray matter; LI, lacunar infarcts; MDt, FW‐corrected mean diffusivity; NAWM, normal‐appearing white matter; NCI, no cognitive impairment; QS, quantitative susceptibility; VaMCI, Vascular Mild Cognitive Impairment; WM, white matter; WMH, white matter hyperintensities.

^a^ Unless otherwise indicated, *p* values were calculated with the Mann–Whitney *U* test.

^b^ Unless otherwise indicated, *p* values were calculated with Student *t* test.

^c^ *p*‐value < 0.05 was considered to be statistically significant.

**p < 0.01, ***p < 0.001.

### 3.4 Associations Between MRI Indices and Attention/Executive Function

**FIGURE 2:**
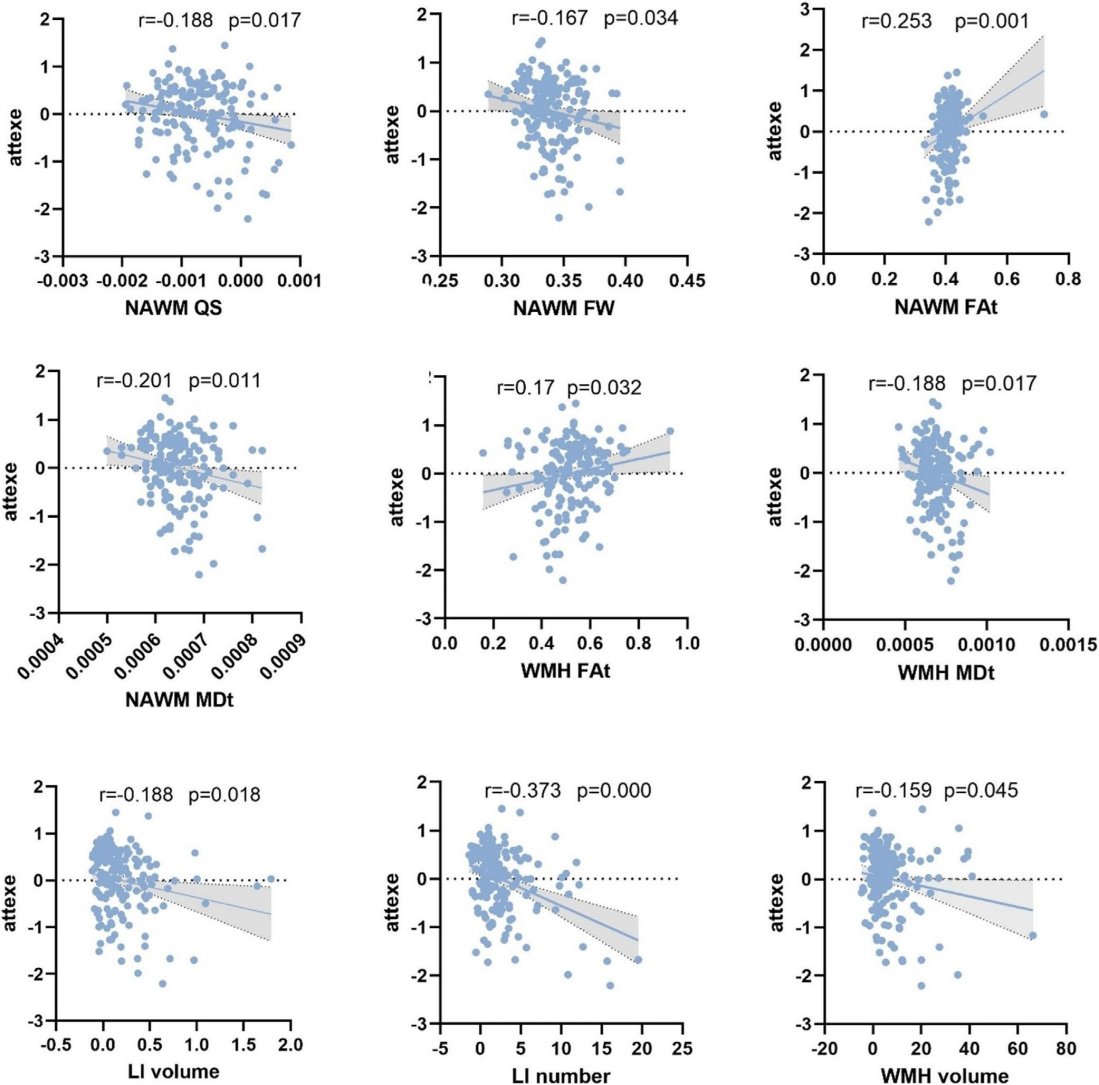
Adjusting for age, gender ratio, education years and relevant risk factors, scatterplots showing the partial correlations between MRI indices and *Z*‐score for attention/executive function. Lines and shaded areas represented the correlation coefficient and 95% confidence interval. attexe, attention/executive function; FAt, FW‐corrected fractional anisotropy; FW, free water; LI, lacunar infarcts; MDt, FW‐corrected mean diffusivity; NAWM, normal‐appearing white matter; QS, quantitative susceptibility; WMH, white matter hyperintensities.

Partial correlation analysis showed that FAt in NAWM and WMH was positively correlated with *Z*‐score for attention/executive function, whereas QS, FW, and MDt in NAWM, MDt in WMH, WMH volume, LI volume, and counts were negatively correlated (Table 5 and Figure 2). No significant associations existed for QS and FW in WMH, GM, and WM volume.

**TABLE 5:** Partial correlations between MRI indices and *Z*‐score for attention/executive function.

MRI indices	*r*	*p*
NAWM QS	−0.188	0.017^a^
NAWM FW	−0.167	0.034^a^
NAWM FAt	0.253	0.001**
NAWM MDt	−0.201	0.011^a^
WMH QS	−0.76	0.343
WMH FW	−1.09	0.171
WMH FAt	0.17	0.032^a^
WMH MDt	−0.188	0.017^a^
WMH volume	−0.159	0.045^a^
GM volume	0.257	0.156
WM volume	0.134	0.094
LI volume	−0.188	0.018^a^
LI counts	−0.373	< 0.001***

Abbreviations: FAt, FW‐corrected fractional anisotropy; FW, free water; GM, gray matter; LI, lacunar infarcts; MDt, FW‐corrected mean diffusivity; NAWM, normal‐appearing white matter; QS, Quantitative susceptibility; WM, white matter; WMH, white matter hyperintensities.

^a^ *p*‐value < 0.05 was considered to be statistically significant.

**p < 0.01, ***p < 0.001.

### 3.5 The Importance of MRI Indices to Attention/Executive Function

**FIGURE 3:**
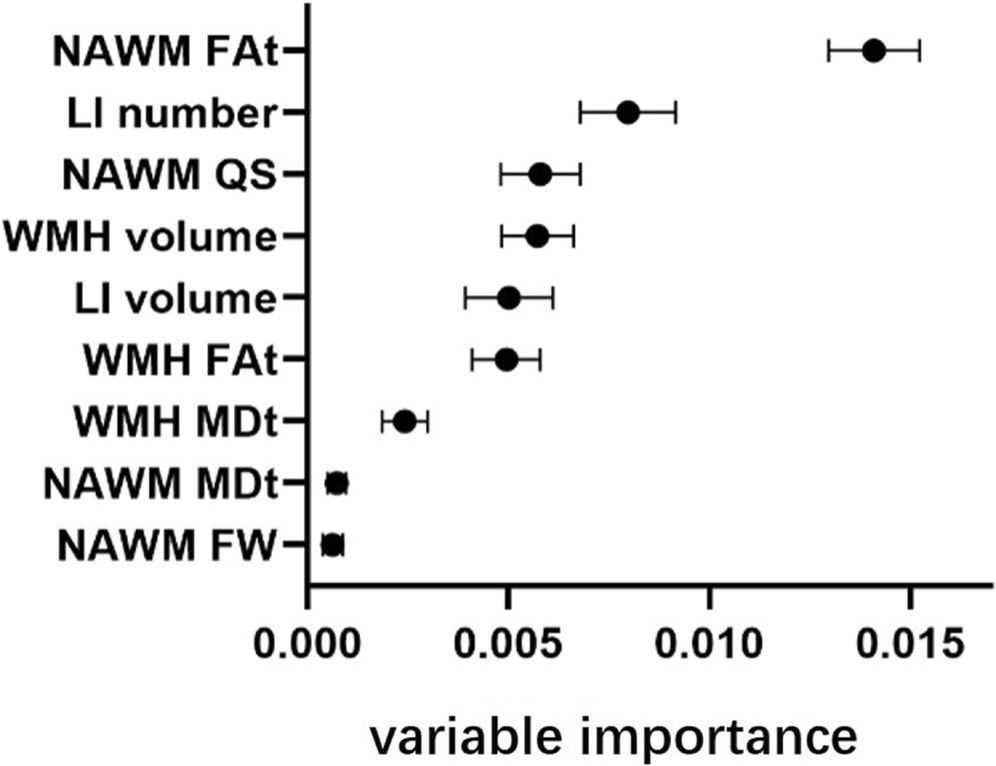
Variable importance of brain MRI indices related to attention/executive function determined by random forest regression. The midpoint of each line referred to the mean value, the starting and ending point of each line represented mean ± SD. FAt, FW‐corrected fractional anisotropy; FW, free water; LI, lacunar infarcts; MDt, FW‐corrected mean diffusivity; NAWM, normal‐appearing white matter; QS, quantitative susceptibility; WMH, white matter hyperintensities.

Based on the results above, the random forest algorithm was applied to determine the importance of MRI indices to attention/executive function. All variables were ranked based on their variable importance. NAWM FAt (variable importance (mean ± SD) = 0.01410 ± 0.00113) had the highest variable importance among MRI indices (Figure 3).

**FIGURE 4:**
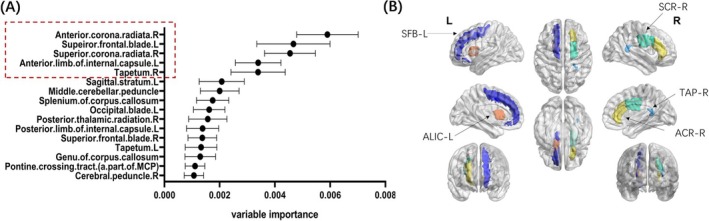
(A) Variable importance of NAWM FAt in part of the 68 white matter tracts related to attention/executive function determined by Random Forest regression. The midpoint of each line referred to the mean, the starting and ending point of each line represented mean ± SD. (B) The 3D illustration of the top five white matter tracts where NAWM FAt values showed highest variable importance for attention/executive function. ACR, anterior corona radiata; ALIC, anterior limb of internal capsule; L, left; *R*, right; SCR, superior corona radiata; SFB, superior frontal blade; TAP, tapetum.

In addition, NAWM FAt in most white matter tracts was related to cognitive decline in attention/executive domain. The complete results of linear regression have been included in the Supporting Information (Table S2). The subsequent random forest analysis identified five WM tracts predominantly influencing attention/executive function, ranked by their variable importance values (mean ± SD): right anterior corona radiata (0.0059 ± 0.00111), left superior frontal blade (0.00467 ± 0.00132), right superior corona radiata (0.00455 ± 0.00092), left anterior limb of the internal capsule (0.00339 ± 0.00082), and right tapetum (0.00339 ± 0.00098). Consistent with prior methodology, we determined the critical inflection point—where the sharp decline in importance values stabilizes—as the threshold for selecting discriminative features [36]. Notably, the variable importance dropped by > 50% (63.77%) between the fifth‐ranked right tapetum (0.00339 ± 0.00098) and the sixth‐ranked left sagittal stratum (0.00207 ± 0.00082). Given this marked reduction, only the top five WM tracts were retained as the most critical brain regions associated with attention/executive function (Figure 4). Of note, we applied a threshold of 0.001 for variable importance to simplify the diagram presentation, while the specific variable importance values for all 68 WM tracts were provided in the supplemental files (Table S3 and Figure S1).

## 4 Discussion

In the current study, we identified three principal findings. First, FAt values in NAWM showed a positive correlation with attention/executive function and demonstrated the highest variable importance among all MRI indices. Second, FAt values in specific white matter tracts, particularly the frontal white matter and thalamic radiation, played a significant role in this relationship. Third, FW and QS values in NAWM, rather than in WMH, were negatively correlated with attention/executive function.

We identified NAWM FAt as a significant MRI index to attention/executive function in CSVD. This finding might be explained by the underlying pathophysiological processes involved in CSVD. CSVD evolves over an extended temporal course, characterized by insidious and progressive deterioration of cognitive capacities [37]. Under ischemic and hypoxic conditions, the integrity of the blood–brain barrier (BBB) is disrupted, with concurrent neuroinflammation and increased BBB permeability observed in CSVD [38, 39]. BBB impairment in CSVD promotes fibrinogen and fluid extravasation into perivascular spaces, inducing inflammatory edema and initiating early microstructural white matter degeneration—notably myelin sheath damage—through interstitial fluid accumulation [40, 41]. FW measurements may potentially result from inflammation‐associated interstitial edema [42]. Myelin loss precedes neuronal axonal degeneration and may exhibit reversibility under specific conditions [43, 44]. QSM enables quantification of myelin content and detection of demyelination in neural tissues, leveraging the distinct magnetic properties of myelin compared to axons [45]. Due to the extremely limited regenerative capacity of axons, damage is often permanent and resistant to effective repair. FA demonstrates significant correlations with axonal density and myelin integrity in the white matter, while FAt represents a more precise measure of axonal structural integrity after correcting for free water [46]. Therefore, advanced‐stage microstructural damage (FAt) critically links to attention/executive dysfunction in CSVD, whereas early‐stage biomarkers (FW, QS) detect reversible edema/demyelination with limited direct cognitive impact.

Our analysis further demonstrated a significant negative association between WMH volume and attention/executive function. However, its variable importance was substantially lower than NAWM FAt. FW and QS alterations within NAWM—rather than WMH—demonstrated significant negative correlations with attention/executive function performance. This finding partially aligns with prior research in normal aging populations, which identified FW increases in both WMH and NAWM as closely associated with processing speed‐dependent executive functions [47]. The previous study primarily investigated samples from the normal aging, whereas our research expands these discoveries to CSVD patients. This observed difference might be interpreted through a pathophysiological lens. Neuroimaging studies have identified a peri‐lesional penumbra surrounding WMH, which exhibited heightened progression risk to WMH conversion [48, 49]. Progressive white matter integrity loss in NAWM preceded conventional MRI‐visible WMH formation, with emerging evidence of perivascular inflammation and neuroinflammatory activity extending beyond MRI‐defined lesion boundaries [50]. WMH demonstrated distinct pathological features including demyelination, neuroinflammatory microglial activation, and reactive astrogliosis, while NAWM exhibited a graded reduction in severity as a function of distance from WMH in CSVD [51]. Our previous longitudinal study showed stable WMH microstructure but dynamic NAWM changes over 1–2 years, partially aligning with our current findings [13]. This suggests that NAWM dynamics may significantly influence cognitive outcomes, indicating that the observed changes in NAWM metrics may have a substantial impact on cognitive function. WMH regions might exhibit near‐complete structural disintegration, whereas NAWM retains partial microarchitectural preservation. Consequently, FAt‐derived microstructural alterations in NAWM, indicative of axonal coherence and myelin integrity, emerge as superior predictors of attention/executive dysfunction compared to both early‐stage FW/QS changes and late‐stage WMH manifestations.

We also found NAWM FAt in several regions, including the right anterior corona radiata, left superior frontal blade, right superior corona radiata, left anterior limb of the internal capsule, and right tapetum, which appeared to play a significant role in attention/executive function. The frontal cortex is widely recognized as the neural substrate for a broad range of higher‐order cognitive functions, including executive control, decision‐making, and social reasoning [52]. The frontal lobe is related to various types of disorders [53, 54]. Neuroimaging evidence identified the frontal aslant tract as critically supporting executive functions, while the right anterior thalamic radiation emerged as a neural mediator of response inhibition [55]. The anterior corona radiata and superior corona radiata are projection fibers within the limbic‐thalamo‐cortical circuitry [56]. The anterior limb of the internal capsule is a portion of the internal capsule mainly composed of thalamic cortical fibers, frontal pontine tract, and connections between the caudate and lenticular nuclei. Our finding further demonstrated the role of the frontal lobe and thalamic radiation in attention/executive function.

This study has several limitations. First, the cross‐sectional design and relatively small sample size limit our ability to fully characterize the temporal dynamics of white matter alterations in CSVD progression. Therefore, further longitudinal investigations with larger cohorts are warranted. Second, the exclusion of vascular dementia patients may restrict the generalizability of our findings. Including vascular dementia cohorts in future studies could provide a more comprehensive understanding of disease progression and inform targeted therapeutic interventions. Third, since pathological examinations are not routinely performed in CSVD patients, our neuroimaging findings were interpreted based on established CSVD pathophysiology, which may require further validation through animal experiments.

## 5 Conclusion

Our findings suggest that in CSVD, microstructural white matter integrity, as indicated by FAt values in NAWM, may predominantly influence attention/executive function. Frontal white matter tracts and thalamic radiation play crucial roles in this context, contributing to the neural circuitry involved in cognitive control and information processing. Our findings may help establish NAWM FAt as a potential neuroimaging biomarker for attention/executive function impairment, providing a valuable tool for early detection and monitoring of cognitive decline in CSVD patients. Furthermore, this research underscores the importance of assessing white matter integrity in clinical evaluations to better predict cognitive outcomes and enhance patient management.

## Author Contributions

**Qingyang Fu:** conceptualization, methodology, formal analysis, visualization, writing original draft. **Yage Qiu:** conceptualization, investigation, data curation, writing original draft. **Ying Hu:** formal analysis, visualization. **Yuanzheng Wang:** methodology, investigation. **Yao Wang:** data curation, formal analysis. **Wentao Hu:** validation and data curation. **Qun Xu:** funding acquisition, resources, project administration. **Yawen Sun:** project administration, funding acquisition, writing – review and editing. **Yan Zhou:** project administration, funding acquisition, writing – review and editing.

## Disclosure

The authors have nothing to report.

## Conflicts of Interest

The authors declare no conflicts of interest.

## Supporting information


Data S1

## Data Availability

Original data included in this study are mainly human MR images and clinical data acquired at our hospital, and is administrated according to the clinical and research ethics policies of the hospital. Therefore, the data could not be acquired from a public repository, but are available from the corresponding author on reasonable and formal requests.
