# Supplementary material for: Free Water‐Corrected Fractional Anisotropy in Normal‐Appearing White Matter as a Potential Neuroimaging Biomarker for Attention and Executive Function Impairment in Cerebral Small Vessel Disease
Source: CNS Neurosci Ther. 2025 Jun 9;31(6):e70475. doi: 10.1111/cns.70475 (PMC12149327; doi:10.1111/cns.70475)
Supplement: Supplementary file 1 — Data S1 [file CNS-31-e70475-s001.docx]

**Supplemental files**

**Table S1. Variable importance of brain MRI indices related to attention/executive function determined by Random Forest regression.**

| **MRI indices** | **Variable importance (mean ± SD）** |
| --- | --- |
| NAWM FAt | 0.01410 ± 0.00113 |
| LI number | 0.00797 ± 0.00119 |
| NAWM QS | 0.00579 ± 0.00099 |
| WMH volume | 0.00572 ± 0.00090 |
| LI volume | 0.00501 ± 0.00109 |
| WMH FAt | 0.00494 ± 0.00084 |
| WMH MDt | 0.00242 ± 0.00056 |
| NAWM MDt | 0.00073 ± 0.00023 |
| NAWM FW | 0.00062 ± 0.00024 |

**Abbreviations:** NAWM = normal-appearing white matter; WMH = white matter hyperintensities; QS = Quantitative susceptibility; FW = free-water; FAt = FW-corrected fractional anisotropy; MDt = FW-corrected mean diffusivity; LI = lacunar infarcts; SD = standard deviation.

**Table S2. Significant linear regression results of attention/executive function regressed on NAWM FAt in specific white matter tracts.**

| **WM tracts** | **Beta** | ***p*-values** |
| --- | --- | --- |
| Anterior corona radiata-L | 3.5871 | < 0.001^***^ |
| Anterior corona radiata-R | 3.9884 | < 0.001^***^ |
| Anterior limb of internal capsule-L | 2.251 | < 0.001^***^ |
| Anterior limb of internal capsule-R | 2.2626 | 0.0032559^**^ |
| Body of corpus callosum | 2.5188 | 0.0034227^*^^*^ |
| Cingulum (cingulate gyrus) -R | 1.7361 | 0.026681^*^ |
| Cingulum- (hippocampus) -L | 2.7263 | 0.025426^*^ |
| Cingulum- (hippocampus) -R | 3.3329 | 0.0029665^**^ |
| External capsule-L | 2.2325 | 0.015653^*^ |
| External capsule-R | 2.5604 | 0.014475^*^ |
| Fornix (column and body of fornix) | 1.4203 | 0.018624^*^ |
| Fornix-L | 4.372 | < 0.001^***^ |
| Fornix-R | 2.5634 | 0.0105^*^ |
| Genu of corpus callosum | 2.932 | < 0.001^***^ |
| Inferior cerebellar peduncle-R | 2.3207 | 0.014933^*^ |
| Inferior frontal blade-L | 2.3858 | 0.0065595^**^ |
| Inferior frontal blade-R | 2.2982 | 0.0053307^**^ |
| Inferior fronto-occipital fasciculus-L | 2.6233 | 0.0011562^**^ |
| Inferior fronto-occipital fasciculus-R | 3.2654 | < 0.001^***^ |
| Medial lemniscus-L | 2.8229 | 0.0025303^**^ |
| Middle cerebellar peduncle | 1.3929 | 0.0077824^**^ |
| Middle frontal blade-L | 2.0897 | 0.0025352^**^ |
| Middle frontal blade-R | 2.7972 | < 0.001^***^ |
| Occipital blade-L | 3.3612 | < 0.001^***^ |
| Occipital blade-R | 3.0503 | < 0.001^***^ |
| Parieto-temporal blade-L | 2.2992 | 0.00229^**^ |
| Parieto-temporal blade-R | 1.5292 | 0.020418^*^ |
| Post-central blade-R | 1.7481 | 0.033095^*^ |
| Posterior corona radiata-L | 2.2386 | 0.0067866^**^ |
| Posterior corona radiata-R | 1.6658 | 0.039407^*^ |
| Posterior limb of internal capsule-L | 3.6262 | < 0.001^***^ |
| Posterior limb of internal capsule-R | 2.9696 | < 0.001^***^ |
| Posterior thalamic radiation-L | 2.426 | 0.0038776^**^ |
| Posterior thalamic radiation-R | 3.0181 | < 0.001^***^ |
| Pre-central blade-R | 1.6892 | 0.048648^*^ |
| Retrolenticular part of internal capsule-L | 1.613 | 0.049752^*^ |
| Retrolenticular part of internal capsule-R | 2.0606 | 0.030655^*^ |
| Sagittal stratum-L | 2.6576 | < 0.001^***^ |
| Sagittal stratum-R | 2.0676 | 0.0098485^**^ |
| Splenium of corpus callosum | 3.1485 | < 0.001^***^ |
| Supeiror frontal blade-L | 2.7932 | < 0.001^***^ |
| Superior cerebellar peduncle-L | 3.1477 | < 0.001^***^ |
| Superior cerebellar peduncle-R | 1.9676 | 0.029732^*^ |
| Superior corona radiata L | 3.4212 | < 0.001^***^ |
| Superior corona radiata-R | 1.7137 | 0.0017648^**^ |
| Superior frontal blade-R | 1.754 | 0.0010738^**^ |
| Superior fronto-occipital fasciculus-L | 3.1016 | 0.0023278^**^ |
| Superior fronto-occipital fasciculus-R | 2.5139 | 0.0039732^**^ |
| Superior longitudinal fasciculus-L | 1.958 | 0.0022955^**^ |
| Superior longitudinal fasciculus-R | 2.1952 | < 0.001^***^ |
| Superior parietal blade-L | 3.5838 | < 0.001^***^ |
| Superior parietal blade-R | 3.0961 | < 0.001^***^ |
| Tapetum-L | 1.6249 | < 0.001^***^ |
| Tapetum-R | 1.5724 | < 0.001^***^ |
| Temporal blade-L | 2.5935 | 0.0092798^**^ |
| Temporal blade-R | 2.7986 | 0.0083812^**^ |

**Abbreviations:** WM = white matter. L = left; R = right.

^*^ *p* -value <0.05 was considered to be statistically significant. ^**^*p* < 0.01, ^***^*p* < 0.001.

**Table S3. Variable importance of NAWM FAt in specific WM tracts related to attention/executive function determined by Random Forest regression (ranked by variable importance).**

| **WM tracts** | **Variable importance (mean ± SD)** |
| --- | --- |
| Anterior corona radiata-R | 0.0059 ± 0.00111 |
| Superior frontal blade-L | 0.00467 ± 0.00132 |
| Superior corona radiata-R | 0.00455 ± 0.00092 |
| Anterior limb of internal capsule-L | 0.00339 ± 0.00082 |
| Tapetum-R | 0.00339 ± 0.00098 |
| Sagittal stratum | 0.00207 ± 0.00082 |
| Middle cerebellar peduncle | 0.002 ± 0.0007 |
| Splenium of corpus callosum | 0.00175 ± 0.00058 |
| Occipital blade-L | 0.00162 ± 0.00057 |
| Posterior thalamic radiation-R | 0.00157 ± 0.00069 |
| Posterior limb of internal capsule-L | 0.00138 ± 0.00058 |
| Superior frontal blade-R | 0.00137 ± 0.00052 |
| Tapetum-L | 0.00133 ± 0.00057 |
| Genu of corpus callosum | 0.0013 ± 0.00055 |
| Pontine crossing tract (a part of MCP) | 0.00111 ± 0.00036 |
| Cerebral peduncle-R | 0.00107 ± 0.00035 |
| Fornix (column and body of fornix) | 0.00099 ± 0.00038 |
| Inferior fronto-occipital fasciculus-L | 0.00079 ± 0.00039 |
| Superior fronto-occipital fasciculus-R | 0.00073 ± 0.00036 |
| Medial lemniscus-L | 0.00072 ± 0.00039 |
| Occipital blade-R | 0.00069 ± 0.00034 |
| Superior fronto-occipital fasciculus-L | 0.00067 ± 0.00036 |
| Cingulum (hippocampus)-R | 0.00065 ± 0.00035 |
| Superior cerebellar peduncle-L | 0.00064 ± 0.00041 |
| Inferior frontal blade-R | 0.0006 ± 0.00027 |
| Parieto-temporal blade-L | 0.0006 ± 0.00035 |
| Posterior limb of internal capsule-R | 0.00058 ± 0.00037 |
| Body of corpus callosum | 0.00057 ± 0.0004 |
| Superior longitudinal fasciculus-R | 0.00054 ± 0.00033 |
| Superior parietal blade-L | 0.00051 ± 0.00039 |
| Anterior corona radiata-L | 0.00049 ± 0.00034 |
| Superior parietal blade-R | 0.00048 ± 0.00035 |
| Inferior cerebellar peduncle-L | 0.00045 ± 0.00019 |
| Superior cerebellar peduncle-R | 0.00043 ± 0.00024 |
| Cingulum (cingulate gyrus)-L | 0.00043 ± 0.00029 |
| Cerebral peduncle-L | 0.00043 ± 0.00022 |
| Anterior limb of internal capsule-R | 0.00042 ± 0.00027 |
| Retrolenticular part of internal capsule-R | 0.00041 ± 0.00023 |
| Uncinate fasciculus-L | 0.0004 ± 0.00022 |
| Medial lemniscus-R | 0.00034 ± 0.00023 |
| Middle frontal blade-L | 0.00034 ± 0.00027 |
| Sagittal stratum-R | 0.00034 ± 0.00019 |
| Inferior frontal blade-L | 0.00034 ± 0.00027 |
| External capsule-L | 0.00033 ± 0.00027 |
| Superior corona radiata-L | 0.00029 ± 0.00025 |
| Fornix-R | 0.00027 ± 0.00023 |
| Superior longitudinal fasciculus-L | 0.00026 ± 0.00023 |
| Corticospinal tract-L | 0.00025 ± 0.00017 |
| Fornix-L | 0.00025 ± 0.00024 |
| Inferior cerebellar peduncle-R | 0.00025 ± 0.0002 |
| Cingulum (hippocampus)-L | 0.00024 ± 0.00017 |
| Middle frontal blade-R | 0.00022 ± 0.00022 |
| Posterior corona radiata-R | 0.00022 ± 0.00019 |
| Corticospinal tract-R | 0.00021 ± 0.00015 |
| Pre-central blade-R | 0.00021 ± 0.00015 |
| Temporal blade-R | 0.00021 ± 0.00017 |
| Pre-central blade-L | 0.0002 ± 0.00013 |
| Posterior thalamic radiation-L | 0.00019 ± 0.00018 |
| Uncinate fasciculus-R | 0.00018 ± 0.00017 |
| Retrolenticular part of internal capsule-L | 0.00018 ± 0.00015 |
| Posterior corona radiata-L | 0.00017 ± 0.00017 |
| Cingulum (cingulate gyrus)-R | 0.00017 ± 0.00016 |
| External capsule-R | 0.00014 ± 0.00014 |
| Post-central blade-L | 0.00014 ± 0.00012 |
| Inferior fronto-occipital fasciculus-R | 0.00013 ± 0.00013 |
| Parieto-temporal blade-R | 0.00012 ± 0.00013 |
| Temporal blade-L | 0.00011 ± 0.0001 |
| Post-central blade-R | 0.00006 ± 0.00008 |

**Abbreviations:** WM = white matter; SD = standard deviation; L = left; R = right.


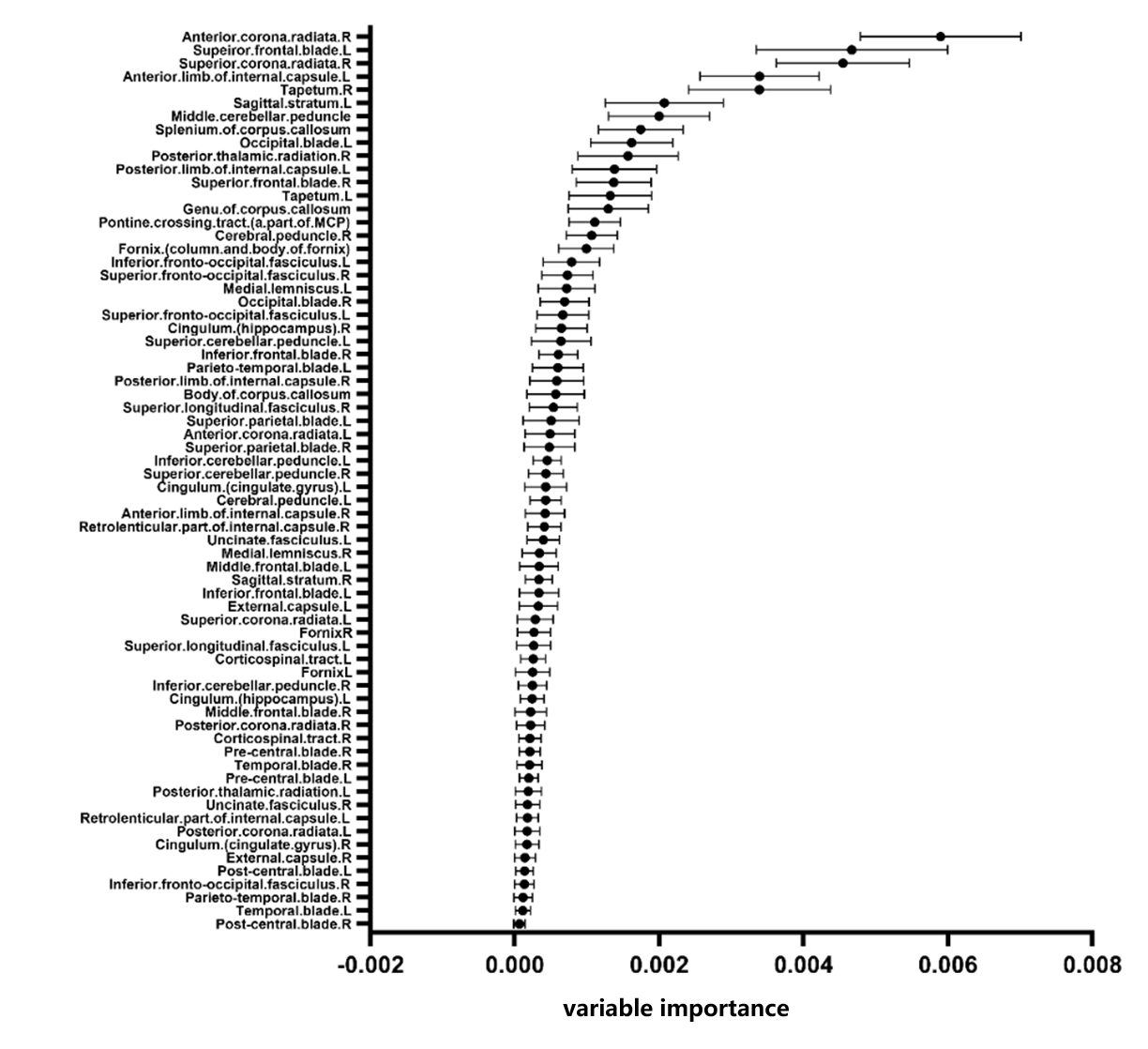
**Figure S1.** Variable importance of NAWM FAt in 68 white matter tracts related to attention/executive function determined by Random Forest regression (ranked by variable importance). The midpoint of each line referred to the mean, the starting and ending point of each line represented mean ± SD.
